# Microbial composition of enigmatic bird parasites: *Wolbachia* and *Spiroplasma* are the most important bacterial associates of quill mites (Acariformes: Syringophilidae)

**DOI:** 10.1002/mbo3.964

**Published:** 2020-03-06

**Authors:** Eliza Glowska, Zuzanna Karolina Filutowska, Miroslawa Dabert, Michael Gerth

**Affiliations:** ^1^ Faculty of Biology Department of Animal Morphology Adam Mickiewicz University in Poznań Poznan Poland; ^2^ Faculty of Biology Molecular Biology Techniques Laboratory Adam Mickiewicz University in Poznań Poznan Poland; ^3^ Department of Biological and Medical Sciences Oxford Brookes University Oxford UK; ^4^ Faculty of Biology Department of Gene Expression Institute of Molecular Biology and Biotechnology Adam Mickiewicz University in Poznań Poznan Poland

**Keywords:** 16S rRNA gene amplicon sequencing, *Bartonella*, birds, *Brucella*, ectoparasites, quill mites

## Abstract

**Background:**

The microbiome is an integral component of many animal species, potentially affecting behavior, physiology, and other biological properties. Despite this importance, bacterial communities remain vastly understudied in many groups of invertebrates, including mites. Quill mites (Acariformes: Syringophilidae) are a poorly known group of permanent bird ectoparasites that occupy quills of feathers and feed on bird subcutaneous tissue and fluids. Most of the known species have strongly female‐biased sex ratio, and it was hypothesized that this is caused by endosymbiotic bacteria. Previously, *Anaplasma phagocytophilum* (Foggie) and a high diversity of *Wolbachia* strains were detected in quill mites via targeted PCR screens. Here, we use an unbiased 16S rRNA gene amplicon sequencing approach to determine other bacteria that potentially impact quill mite biology.

**Results:**

We performed 16S rRNA gene amplicon sequencing of 126 quill mite individuals from eleven species parasitizing twelve species (four families) of passeriform birds. In addition to *Wolbachia*, we found *Spiroplasma* as potential symbiont of quill mites. Consistently, high *Spiroplasma* titers were only found in individuals of two mite species associated with finches of the genus *Carduelis*, suggesting a history of horizontal transfers of *Spiroplasma* via the bird host. Furthermore, there was evidence for *Spiroplasma* negatively affecting *Wolbachia* titers. We found no evidence for the previously reported *Anaplasma* in quill mites, but detected sequences of high similarity to the potential pathogens *Brucella* and *Bartonella* at low abundances. Other amplicon sequence variants (ASVs) could be assigned to a diverse number of bacterial taxa, including several that were previously isolated from bird skin. Further, many frequently found ASVs were assigned to taxa that show a very broad distribution with no strong prior evidence for symbiotic association with animals. We interpret these findings as evidence for a scarcity of resident microbial associates (other than inherited symbionts) in quill mites.

## Graphical abstract

We found a diverse, but relatively uniform set of bacterial taxa within quill mites that include arthropod endosymbionts, potential pathogens, and bird associated bacteria. The importance of most of these microbes for quill mite biology is unclear, but abundances and distribution patterns suggest that Spiroplasma and Wolbachia are the most important quill mite associates.

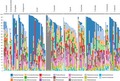

## 1 INTRODUCTION

There is abundant evidence that microbial taxa are an essential component of many animal species (McFall‐Ngai et al., 2013). Bacteria‐encoded traits may significantly impact host phenotypes, for example, through providing essential nutrients (Duron et al., 2018; Hosokawa, Koga, Kikuchi, Meng, & Fukatsu, 2010), defending against pathogens (Ballinger & Perlman, 2017; King et al., 2016), but also affecting ecological features of their hosts, such as mate choice (Sharon et al., 2010) and life history traits (Laughton, Fan, & Gerardo, 2013). Because of their potential importance in understanding the biology of many organisms, the number of microbiome studies has been soaring (Hird, 2017). This popularity is owed to methodological advances (high‐throughput sequencing technologies) allowing comprehensive investigation of the microbial communities (Ji & Nielsen, 2015), but also to the decreasing costs of these approaches (Sboner, Mu, Greenbaum, Auerbach, & Gerstein, 2011). However, the main focus on microbiome studies so far has been vertebrates (Colston & Jackson, 2016) and invertebrates of medical, veterinary, or economical importance. For example, in mites, microbiome studies have been conducted on the stored product pests (Erban et al., 2016; Hubert, Kopecky, Nesvorna, Perotti, & Erban, 2016; Hubert et al., 2019), house dust mites (Chan et al., 2015; Oh, Ishii, Tongu, & Itano, 1986; Valerio, Murray, Arlian, & Slater, 2005), and mites transmitting pathogens, such as sheep scab mites (Hogg & Lehane, 1999), red poultry mites (Hubert et al., 2017; Moro, Thioulouse, Chauve, & Zenner, 2011), and the honey bee parasite *Varroa* (Hubert, Kamler, et al., 2016).

In the present study, we focus on quill mites (Acariformes: Syringophilidae). These obligatory bird ectoparasites live and reproduce inside the quills of feathers where they feed on subcutaneous fluids (lymph, blood). Quill mite dispersion has been observed on the same individual (from infected to uninfected feathers), between individuals of the same species (e.g., from parents to hatchings) and occasionally by transfer between gregarious bird species (Casto, 1974a,1974b; Kethley, 1970, 1971). This mode of feeding and dispersion makes quill mites potential vectors for bacterial pathogens, similar to ticks or lice (Azad & Beard, 1998). However, only two bacterial taxa were recorded in quill mites so far: (a) *Anaplasma phagocytophilum* (Foggie) (Alphaproteobacteria, Rickettsiales) was detected in two quill mite species from three bird species (Skoracki et al., 2006); (b) multiple genetically distinct lineages of *Wolbachia* (Alphaproteobacteria, Rickettsiales) were found in five species of quill mites (Glowska, Dragun‐Damian, Dabert, & Gerth, 2015). As these studies were targeted PCR screens, it remains unclear what other bacteria populate quill mites. Furthermore, the importance of quill mites for bird pathogen dynamics is not known.

To address these questions, we here assess the bacterial composition of 126 quill mite individuals encompassing eleven species with a more unbiased 16S rRNA gene amplicon sequencing approach. We find that the symbionts *Wolbachia* and *Spiroplasma* are among the most commonly associated taxa with quill mites. Other taxa include bacteria that were previously found in association with arthropods and bacteria with a very broad distribution. Strikingly, neither quill mite taxonomy nor bird host taxonomy significantly influences bacterial composition in quill mites. Furthermore, we find that despite the detection of *Bartonella* and *Brucella*, quill mites do not seem to be major pathogen vectors in birds.

## 2 MATERIAL AND METHODS

### 2.1 Animal collection and DNA extraction

A summary of collected quill mite species and their bird hosts can be found in Table 1. All quill mites used in this study were collected in Kopan, Poland, during spring migration of birds monitored by the Bird Migration Research Station, University of Gdansk, April 2009. One secondary flight feather was analyzed from each bird specimen and dissected under a stereo microscope (Olympus ZS30). Individual mites were washed twice and preserved in 96% ethanol, and total genomic DNA was extracted from single specimens using DNeasy Blood & Tissue Kit (Qiagen GmbH), as described previously (Dabert, Ehrnsberger, & Dabert, 2008). This procedure left the exoskeletons intact, and the specimens were subsequently mounted on microscopic slides in Faure medium and determined using the key from Skoracki, Spicer, and Oconnor (2016). All morphological observations were carried out with an Olympus BH2 microscope with differential interference contrast (DIC) optics and a camera lucida. All DNA samples and corresponding voucher specimens are deposited in the collection of the Department of Animal Morphology, Faculty of Biology, Adam Mickiewicz University in Poznan, Poland. To identify potential contaminants, in addition to sequencing a negative control alongside all samples, we further extracted DNA from reagents and materials commonly used in the laboratory this work was carried out in. One library each was created from extraction buffer (ALT), millipore water, microscope swabs, pipette swabs, and swabs of other equipment (pincettes, scalpels, benches, etc). These five libraries were processed and sequenced separately from the other samples, but by using identical procedures.

**Table 1:** Overview of quill mites sampled for the study with average abundance of *Spiroplasma* and *Wolbachia*

Quill mite species	Bird host species (common name)	Number of bird individuals	Number of mite individuals	Average *Spiroplasma* abundance %	Average *Wolbachia* abundance %
*Syringophilopsis kirgizorum*	*Carduelis carduelis* (European goldfinch)	2	9	0.43	2.60
*Torotrogla cardueli*	*Carduelis carduelis* (European goldfinch)	1	6	55.10	0.
*Syringophilopsis kirgizorum*	*Carduelis chloris* (European greenfinch)	1	2	62.00	1.45
*Aulobia cardueli*	*Carduelis flammea* (Common redpoll)	1	2	0.00	0.06
*Aulobia cardueli*	*Carduelis spinus* (Eurasian siskin)	1	4	0.34	1.53
*Torotrogla cardueli*	*Carduelis spinus* (Eurasian siskin)	1	13	13.70	2.41
*Torotrogla rubeculi*	*Erithacus rubecula* (European robin)	3	12	0.14	5.92
*Syringophilopsis fringillae*	*Fringilla coelebs* (Common chaffinch)	1	6	0.00	6.59
*Torotrogla gaudi*	*Fringilla coelebs* (Common chaffinch)	2	16	0.55	0.01
*Torotrogla lusciniae*	*Luscinia luscinia* (Thrush nightingale)	1	7	0.35	0.51
*Torotrogla lusciniae*	*Luscinia svecica* (Bluethroat)	1	1	1.07	0.61
*Torotrogla modularis*	*Prunella modularis* (Dunnock)	1	4	0.00	0.10
*Syringophiloidus parapresentalis*	*Turdus iliacus* (Redwing)	1	3	0.00	0.21
*Syringophilopsis turdi*	*Turdus iliacus* (Redwing)	3	15	0.00	5.92
*Torotrogla merulae*	*Turdus merula* (Common blackbird)	3	13	0.00	4.19
*Syringophilopsis turdi*	*Turdus philomelos* (Song thrush)	1	4	0.00	12.10
*Torotrogla merulae*	*Turdus philomelos* (Song thrush)	2	9	0.00	3.80

### 2.2 Library preparation and sequencing

The V4 hypervariable region of 16S rRNA gene was amplified using PCR primers V4F (GATCAGCAGCCGCGGTAATA) (developed in this study) and V4R (GGACTACCAGGGTATCTAA) (Therese, Anand, & Madhavan, 1998) fused with indexes and Ion Torrent adapters (Table 2). For the PCRs, each 10 µl sample was prepared in two technical replicates containing 2 µl HOT FIREPol Blend Master Mix (Solis BioDyne), 0.25 µM of each double‐indexed fusion primer, and about 1 ng of template DNA. The fusion PCR regime used was 12 min at 95°C, 40 cycles of 15 s at 95°C, 30s at 58°C, 30s at 72°C, and a final 7 min at 72°C. After PCR, all samples were pooled, size‐selected on a 3% agarose gel, purified using the QIAquick Gel Extraction Kit (Qiagen), and quantified on a 2,200 TapeStation (Agilent Technologies, Inc.). Clonal template amplification on Ion Sphere Particles (ISPs) was performed using the Ion Torrent One Touch System II and the Ion PGM™ Hi‐Q™ View OT2 Kit with regard to manufacturer's instructions. Sequencing of the templated ISPs was conducted on the Ion 318™ Chip with the use of Ion PGM™ Hi‐Q™ View Sequencing Kit and Ion PGM system (Ion Torrent, Thermo Fisher Scientific, Inc.) at Molecular Biology Techniques Laboratory, Faculty of Biology, AMU. All reads resulting from the sequencing are available under NCBI BioProject accession PRJNA482380.

**Table 2:** Fusion PCR primers sequences used in this study. Unique random barcode sequences are highlighted in bold

Primer name	Primer sequence
V4FA49	CCATCTCATCCCTGCGTGTCTCCGACTCAGTCCTAACATAACGATCAGCAGCCGCGGTAATA
V4FA50	CCATCTCATCCCTGCGTGTCTCCGACTCAGCGGACAATGGCGATCAGCAGCCGCGGTAATA
V4FA51	CCATCTCATCCCTGCGTGTCTCCGACTCAGTTGAGCCTATTCGATCAGCAGCCGCGGTAATA
V4FA52	CCATCTCATCCCTGCGTGTCTCCGACTCAGCCGCATGGAACGATCAGCAGCCGCGGTAATA
V4FA53	CCATCTCATCCCTGCGTGTCTCCGACTCAGCTGGCAATCCTCGATCAGCAGCCGCGGTAATA
V4FA54	CCATCTCATCCCTGCGTGTCTCCGACTCAGCCGGAGAATCGCGATCAGCAGCCGCGGTAATA
V4FA55	CCATCTCATCCCTGCGTGTCTCCGACTCAGTCCACCTCCTCGATCAGCAGCCGCGGTAATA
V4FA56	CCATCTCATCCCTGCGTGTCTCCGACTCAGCAGCATTAATTCGATCAGCAGCCGCGGTAATA
V4FA57	CCATCTCATCCCTGCGTGTCTCCGACTCAGTCTGGCAACGGCGATCAGCAGCCGCGGTAATA
V4FA58	CCATCTCATCCCTGCGTGTCTCCGACTCAGTCCTAGAACACGATCAGCAGCCGCGGTAATA
V4FA59	CCATCTCATCCCTGCGTGTCTCCGACTCAGTCCTTGATGTTCGATCAGCAGCCGCGGTAATA
V4FA60	CCATCTCATCCCTGCGTGTCTCCGACTCAGTCTAGCTCTTCGATCAGCAGCCGCGGTAATA
V4FA61	CCATCTCATCCCTGCGTGTCTCCGACTCAGTCACTCGGATCGATCAGCAGCCGCGGTAATA
V4FA62	CCATCTCATCCCTGCGTGTCTCCGACTCAGTTCCTGCTTCACGATCAGCAGCCGCGGTAATA
V4FA63	CCATCTCATCCCTGCGTGTCTCCGACTCAGCCTTAGAGTTCGATCAGCAGCCGCGGTAATA
V4FA64	CCATCTCATCCCTGCGTGTCTCCGACTCAGCTGAGTTCCGACGATCAGCAGCCGCGGTAATA
V4RP165	CCTCTCTATGGGCAGTCGGTGATGTCGCTCCAATGGACTACCAGGGTATCTAA
V4RP786	CCTCTCTATGGGCAGTCGGTGATGAGGAACTGGTGGACTACCAGGGTATCTAA
V4RP555	CCTCTCTATGGGCAGTCGGTGATGAAGTTGTAGTGGACTACCAGGGTATCTAA
V4RP333	CCTCTCTATGGGCAGTCGGTGATGATCCAGGCATGGACTACCAGGGTATCTAA
V4RP734	CCTCTCTATGGGCAGTCGGTGATGCGGTTGGCTTGGACTACCAGGGTATCTAA
V4RP299	CCTCTCTATGGGCAGTCGGTGATGCCAGAAGAATGGACTACCAGGGTATCTAA
V4RP564	CCTCTCTATGGGCAGTCGGTGATGACGACAAGGTGGACTACCAGGGTATCTAA
V4RP280	CCTCTCTATGGGCAGTCGGTGATGACCATTAGATGGACTACCAGGGTATCTAA
V4RP684	CCTCTCTATGGGCAGTCGGTGATGAAGAATTCATGGACTACCAGGGTATCTAA
V4RP290	CCTCTCTATGGGCAGTCGGTGATGACCACTCGGTGGACTACCAGGGTATCTAA
V4RP178	CCTCTCTATGGGCAGTCGGTGATGCCGGTAGAATGGACTACCAGGGTATCTAA
V4RP322	CCTCTCTATGGGCAGTCGGTGATGTAGCTTAGGTGGACTACCAGGGTATCTAA
V4RP266	CCTCTCTATGGGCAGTCGGTGATGAATTACAGATGGACTACCAGGGTATCTAA
V4RP388	CCTCTCTATGGGCAGTCGGTGATGTATGGCCGATGGACTACCAGGGTATCTAA
V4RP591	CCTCTCTATGGGCAGTCGGTGATGATCGACTTATGGACTACCAGGGTATCTAA
V4RP357	CCTCTCTATGGGCAGTCGGTGATGTTCATCTCGTGGACTACCAGGGTATCTAA

### 2.3 Read processing and statistical analyses

Reads were trimmed of adaptors and primer sites by using cutadapt version 1.16 (Martin, 2011). The remaining reads were dereplicated, denoised, and chimeras eliminated using the DADA2 package version 1.8 (Callahan et al., 2016) within the R statistical programming environment (R Core Team 2015). Taxonomic assignment of the ASVs (amplicon sequence variants), to species level where possible, was also performed within DADA2 using the SILVA database version 132 (Quast et al., 2013). Next, contaminant taxa were identified from the sequenced extraction control using the “prevalence” algorithm implemented in the R package decontam (Davis, Proctor, Holmes, Relman, & Callahan, 2018). Further potential contaminants were identified by processing the five libraries derived from reagents and materials as described, and then excluding all ASVs that were found in any of these control libraries from subsequent analyses.

To reduce the impact of ASVs with very low abundance, we removed all ASVs that were present in only a single sample and also discarded ASVs from bacterial phyla that only occurred once in total. To account for potential biases between samples with uneven sequencing depth, all read counts from the remaining samples were rarefied to the read depth of the sample with the lowest read number. An overview of how our filtering steps affected ASV counts can be found in Table 3. All subsequent statistical analyses were done on log‐transformed read counts. Because the symbionts *Wolbachia* and *Spiroplasma* were dominant in some of the samples, we excluded all ASVs corresponding to these taxa prior to statistical comparisons between groups. First, we plotted the abundance of the most frequently found bacterial families using the R packages phyloseq and ggplot2 (McMurdie & Holmes, 2013; Wickham, 2009). Next, ordination analyses were performed with phyloseq using Bray distances and non‐metric multidimensional scaling (NMDS). Differences in abundances of particular taxa between groups (quill mite species, bird host species, developmental stage, *Wolbachia* positive and negative samples) were determined with Kruskal–Wallis rank sum tests, and *p*‐values were adjusted to these multiple comparisons to control for the false discovery rate (Benjamini & Hochberg, 1995). These tests were done separately for differences in abundance of bacterial phyla, orders, families, and genera. Furthermore, we calculated Jensen–Shannon distances between the aforementioned groups and used adonis tests (analysis of variance using distance matrices) implemented in the package vegan (Dixon, 2003) to test if they differed significantly. A phyloseq object file containing all data used in the described analyses is available at ://doi.org/10.5281/zenodo.3475151.

**Table 3:** Overview on the impact of filtering and decontamination on the number of retained ASVs and samples in this study. For details on each of the steps, please refer to the materials and methods section

Step	#ASVs	#samples
Unfiltered	4,309	152
Decontamination with “decontam” prevalence method	4,275	152
Remove phyla with only one representative	4,248	152
Removal of ASVs present in only one sample	1,016	152
Removal of samples with very low read count (<3,500)	1,016	126
Rarefy to even depth	983	126
Remove all taxa present in sequenced laboratory reagents and equipment	912	126

## 3 RESULTS

**Figure 1:**
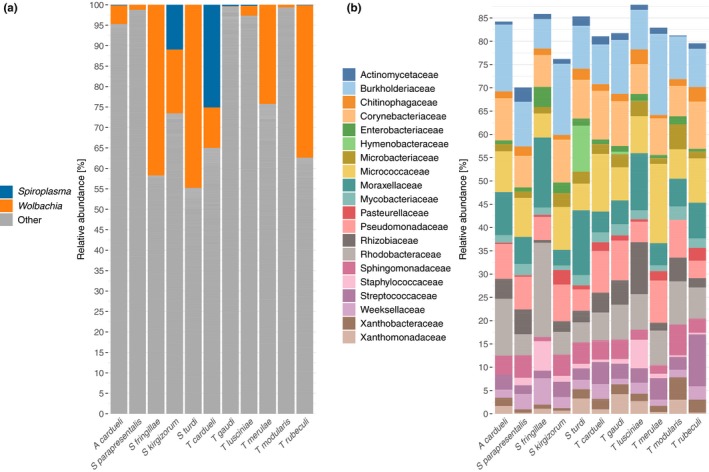
Overview of the bacterial taxa detected in quill mites. (a) Relative abundances for the endosymbionts *Spiroplasma* and *Wolbachia.* (b) Relative proportions of the 20 most abundantly found bacterial families in a dataset without the symbionts *Spiroplasma* and *Wolbachia*. For (a) and (b), each bar represents the averaged abundances across all samples of a single species. Height of stacks represents relative abundances of each taxon. For abundance plots of all samples, please refer to Appendix Figure A1

We have investigated the microbial composition of 126 individuals belonging to eleven quill mite species that parasitize twelve bird host species of passeriform birds. Amplicon sequencing of the v4 region of the 16S rRNA gene on an IonTorrent resulted in 1,582,340 reads, with 9,426 reads/sample on average (4,616–20,231). After processing of reads (quality filtering, denoising, annotation, low abundance filtering, rarefying, decontamination—see methods for details), 912 ASVs were retained. Among the most abundant bacterial genera found in quill mites were *Wolbachia* and *Spiroplasma* (Figure 1a). Because these symbionts were not equally abundant across samples and might thus bias estimates of bacterial composition, they were excluded from the subsequent analyses.

**Figure 2:**
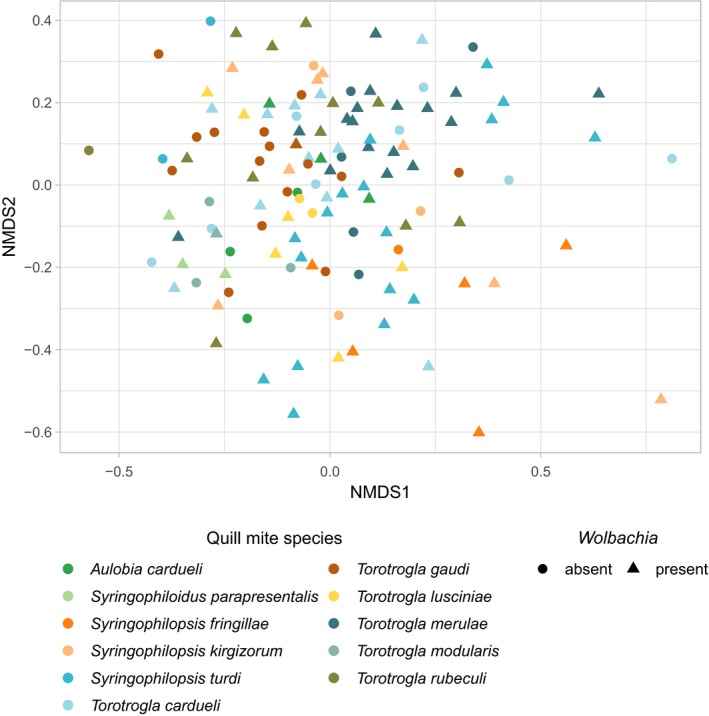
Similarity of quill mite microbiota without the endosymbionts *Spiroplasma* and *Wolbachia* **.** Ordination analysis is based on non‐metric multidimensional scaling (NMDS) and bray distances. Log‐transformed abundances were analyzed. Colors of the dots represent different quill mite species from which the samples were isolated. Shape of the dots stand for *Wolbachia* infection status

**Figure 3:**
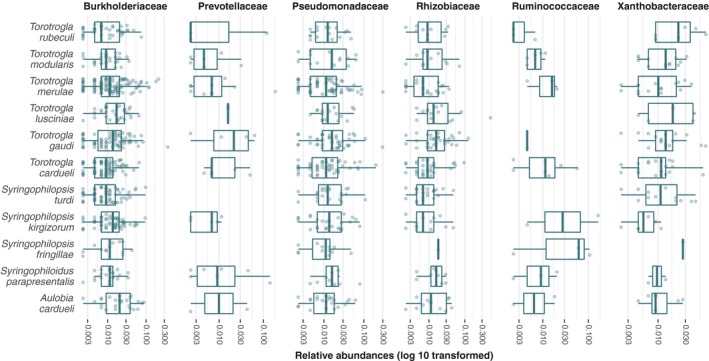
Abundance of five bacterial families that were found to be differentially abundant between quill mite species analyzed. Counts for the symbionts *Wolbachia* and *Spiroplasma* were excluded

**Figure A1:**
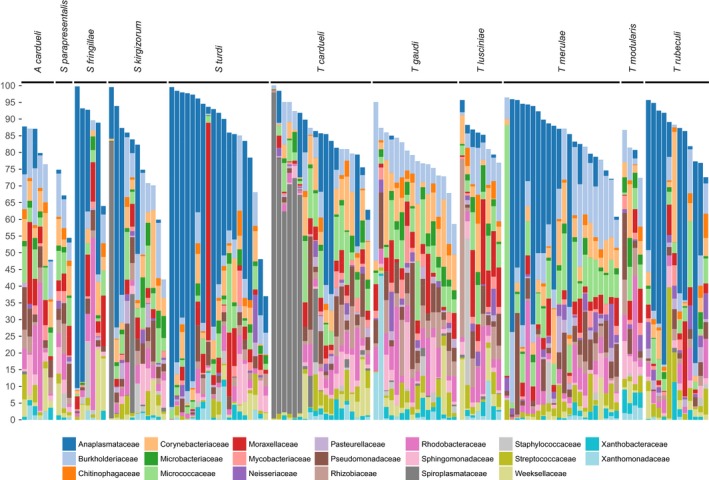
Overview of the bacterial taxa detected in quill mites. Bar plots show the 20 most abundantly found bacterial families. Each stacked bar represents one sample, and the samples are ordered by quill mite species. Height of stacks represents relative abundances of each taxon. Note that all Anaplasmataceae ASVs are *Wolbachia*, and all Spiroplasmataceae ASVs are *Spiroplasma*

Bar plots of ASV abundance and ordination analyses with this filtered dataset revealed that the bacterial composition was relatively uniform across samples, and no clear differentiation between samples extracted from different mite species, or between *Wolbachia* positive and negative a1 samples could be observed (Figures 1b, 2, see also Appendix Figure A1). However, when trying to identify differential abundance patterns of microbial composition between groups using analysis of variances, we found that bacterial composition was more similar between samples from the same quill mite species or genus and bird host species or genus than expected by chance (*p* < .01). Furthermore, six bacterial families were found to be differentially abundant between quill mite species with a Kruskal–Wallis test (*p* < .01, Figure 3), one of which (Xanthobacteraceae) was also found to differ between samples of different bird host genera.

Out of 912 detected ASVs, the 10 most abundantly encountered genera were *Micrococcus*, *Corynebacterium*, *Acinetobacter*, *Streptococcus*, *Burkholderia*, *Phyllobacterium*, *Ralstonia*, *Mycobacterium*, *Paracoccus*, and *Sediminibacterium* (for a full list of ASVs, see Appendix Table A1 at ://doi.org/10.5281/zenodo.3475151). None of these taxa seemed dominant in any sampled group (based on mite or bird taxonomy), and the 20 most abundant families made up similar proportions of the total ASVs across samples (Figure 1b). Other notable findings were the pathogens *Brucella* which was detected in 20 samples with an average abundance of 1.3%, and *Bartonella* which was found in two samples at 1.8% and 0.7% relative abundance, respectively.

**Figure 4:**
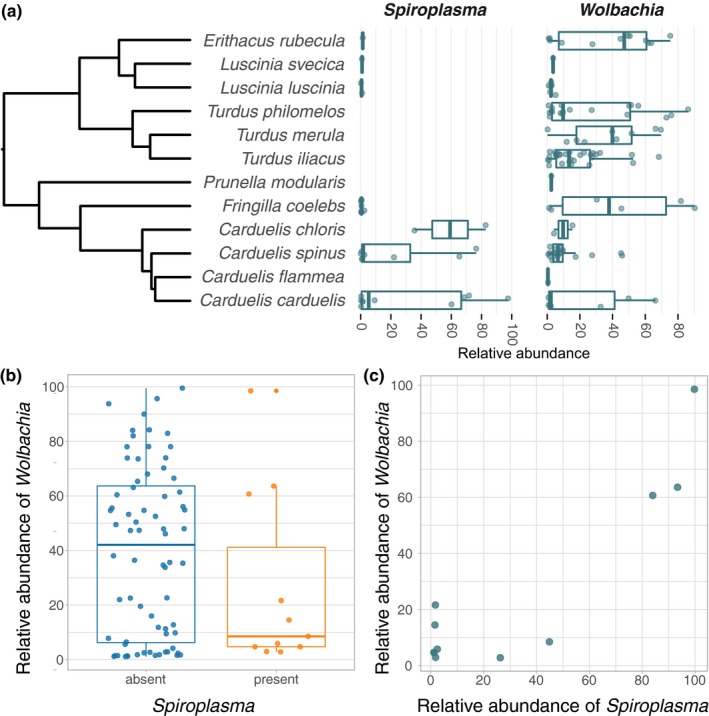
Relative abundances of the endosymbionts *Wolbachia* and *Spiroplasma* in quill mite samples. (a) Abundances for all samples that are *Spiroplasma* and/or *Wolbachia* positive, sorted by bird host species from which the quill mites were isolated. Bird species phylogeny was taken from Jetz, Thomas, Joy, Hartmann, and Mooers (2012; https://birdtree.org/). (b) Relative *Wolbachia* abundances in samples with and without *Spiroplasma*. (c) Correlation of *Wolbachia* and *Spiroplasma* abundances for samples in which both symbionts were present. For (b) and (c), only samples with abundances ≥ 1% are shown. Also, to avoid biases of abundance estimates based on a single dominant taxon, *Spiroplasma* and *Wolbachia* abundances shown in (b) and (c) were corrected for the presence of the other endosymbiont, that is, *Wolbachia* and *Spiroplasma* abundance is plotted relative to the non‐symbiont microbiome. For uncorrected *Spiroplasma* and *Wolbachia* abundances for all samples, please refer to Table 1 and Appendix Table A1

As opposed to the general trend in the microbiome composition data, there was strong evidence for differential abundance of the symbionts *Wolbachia* and *Spiroplasma* between the bird hosts from which the mites were collected. For example, high *Spiroplasma* titers were only observed in two mite species collected from the host genus *Carduelis* (Figures 1a, 4a, Table 1). Further, although *Wolbachia* was present in mites sampled from all bird hosts, it was especially prevalent in mites collected from birds of the genera *Turdus*, *Erithacus*, and *Fringilla*. On contrast, it was absent or at very low titers in mites parasitizing *Luscinia* sp. (Figure 4a, Table 1). On average, the abundance of *Wolbachia* was lower in samples that also contained *Spiroplasma* (Figure 4b). Notably, this was not an effect of *Spiroplasma* presence reducing the amount of available reads for *Wolbachia* (Figure 4b). For mites harboring both symbionts (eleven samples in total), we found that the abundances for *Wolbachia* and *Spiroplasma* are positively correlated (Figure 4c).

## 4 DISCUSSION

### 4.1 Origin of microbial DNA in quill mites

We have sequenced microbial taxa from quill mites, an enigmatic group of bird ectoparasites. The taxa detected through 16S rRNA gene sequencing may be (a) resident symbionts of quill mites, (b) environmentally acquired, transient bacteria, or (c) contaminants from reagents and materials. Each of these options comes with a number of assumptions that can be tested with our data.

For “true,” resident symbionts, one would expect high abundances in at least some of the investigated hosts, presence in all individuals of a host species, and specialization of the symbionts, measurable as genetic differentiation between the symbionts of different host taxa. For example, all honey bees (*Apis* sp.) harbor seven core gut microbial taxa, five of which are present in other corbiculate bees, and two that are not found anywhere else (Kwong, Medina, & Koch, 2017). The composition of these taxa is correlated with phylogenetic distances in this clade of bees, suggesting long‐term association of the microbes with bees. In our dataset, *Wolbachia* and *Spiroplasma* are the most likely candidates for true symbiotic associations. Both bacteria are known as endosymbionts from other arthropods and are unable to permanently live outside their hosts (Anbutsu & Fukatsu, 2011; Makepeace & Gill, 2016). Further, we document a very high abundance of these taxa in at least some of the investigated samples (Figure 4), which is in line with the assumptions above. In a previous study, *Wolbachia* strains of quill mites were investigated with a multi‐locus approach and it has been shown that quill mite associated strains are genetically very different to any other *Wolbachia* strains described so far (Glowska et al., 2015). Here, we have found eight different ASVs annotated as *Wolbachia*, each of which is 100% identical to at least one *Wolbachia* sequence previously isolated from quill mites. For *Spiroplasma*, we found a single ASV that is only 92% identical to the next closest match in the Silva database. This implies that *Spiroplasma* in quill mites might be genetically distinct from *Spiroplasma* of other arthropods, as is the case for *Wolbachia*. However, sequencing data of more loci are needed to establish the phylogenetic placement of *Spiroplasma* from quill mites.
For environmentally acquired, transient taxa, the expectation is that the microbial composition detected in the host reflects the microbial composition of its environment stronger than it reflects host‐specific factors. For example, the gut microbiome of some caterpillars is dominated by bacteria that derive from their food, evidenced by similar bacterial composition of leave surfaces and caterpillar feces (Hammer, Janzen, Hallwachs, Jaffe, & Fierer, 2017). Quill mites live permanently within feather quills of their bird hosts; hence, one might expect to find similar taxa in feathers or on bird skin as in quill mites. Unfortunately, none of the bird hosts sampled in our study was investigated previously with regard to resident skin or feather microbes. One of the most comprehensive feather microbiome studies was performed in the Dark‐eyed Juncos *Junco hyemalis* (L.) and revealed that feathers of these birds harbor bacteria commonly occurring in the soil and phyllosphere (*Brevundimonas*, *Methylobacterium*, and *Sphingomonas*), as well as potential plant pathogens (e.g., *Sphingomonas*, *Microbacterium*, *Curtobacterium*, and *Rathayibacter*) (Dille, Rogers, & Schneegurt, 2016). All of these taxa were also found in our study, suggesting a potential environmental determinant of the bacterial composition we observed in quill mites. Furthermore, many of the core bacterial families described in bird skin microbiome studies were also found in quill mites (e.g., Pseudomonadaceae, Methylobacteriaceae, Corynebacteriaceae, Moraxellaceae, Mycobacteriaceae, Leuconostocaceae, Staphylococcaceae, Lactobacillaceae, Micrococcaceae, Streptococcaceae, Enterobacteriaceae, Sphingomonadaceae, Neisseriaceae, Xanthomonadaceae, and Weeksellaceae) (Engel et al., 2018; Pearce, Hoover, Jennings, Nevitt, & Docherty, 2017). Despite these similarities, and some statistical support for bird hosts shaping the microbiome community in our study, the lack of clustering in ordination analysis indicates that environment is not the major determining factor of quill mite microbiome composition.
It is important to consider that contaminants from reagents and kits may significantly impact microbiome composition estimates, especially when using low biomass samples such as quill mites (de Goffau et al., 2018; Łukasik et al., 2017; Salter et al., 2014). This is problematic in any microbiome study and is very difficult to exclude with certainty. Here, we removed contaminants statistically in silico based on the microbial composition of the sequenced extraction control (Davis et al., 2018). Further, we removed all ASVs present in independently sequenced controls derived from reagents and equipment commonly used in the laboratory where this study was performed (see methods for details). However, a number of ASVs we recovered correspond to common kit contaminants in 16S rRNA microbiome studies (e.g., *Ralstonia*, *Kocuria*), human skin bacteria (*Corynebacterium*), or ubiquitous taxa with no strong evidence for symbiotic associations with arthropods (*Pseudomonas*, *Acinetobacter*). These taxa might constitute true associates, but we cannot exclude the possibility that they originate from contaminating sources.

In summary, we found a diverse range of bacteria associated with quill mites. The lack of differentiation between different mite species or between species collected from different bird hosts leads us to conclude that there are no strong associations with typical gut bacteria as observed in other arthropods. However, we cannot exclude that we missed such potential associates due to the limited amount of DNA that can be extracted from the minute hosts.

### 4.2 Exchange of bacteria via bird hosts

Due to their ectoparasitic life style with occasional host switching, quill mites have the potential to transmit bacteria between their hosts. Here, we detected two pathogenic microbes that might be important in that respect: *Brucella* and *Bartonella*. *Brucella* is the agent of brucellosis, which is considered to be the most widespread zoonotic infection (Pappas, Papadimitriou, Akritidis, Christou, & Tsianos, 2006). Several *Brucella* species are a human health threat, and people typically become infected through contact with domesticated *Brucella* infected animals, such as goats, sheep, or swine (Young, 2006). However, several blood‐sucking arthropods, such as ticks and lice, are regarded as possible vectors for *Brucella* (Neglia et al., 2013; Pritulin, 1954; Rementzova, 1966). To our knowledge, there is no data indicating that Acari other than ticks are natural *Brucella* carriers. It was hypothesized that birds and other wild animals act as natural reservoirs for *Brucella* (Zheludkov & Tsirelson, 2010), which is in line with our finding of this bacterium in bird ectoparasites. The importance of quill mites in spreading *Brucella* between bird species remains to be assessed, but its prevalence (21/126 investigated individuals, 8 different mite species) suggests its finding is of potential importance in understanding this pathogen's dynamics.

*Bartonella* are gram‐negative bacteria that are typically transmitted by blood‐sucking arthropods and are infectious in mammalian hosts (Billeter, Levy, Chomel, & Breitschwerdt, 2008; Klangthong et al., 2015; Reeves, Nelder, Cobb, & Dasch, 2006). There are also reports on *Bartonella* incidence in birds (Ebani, Bertelloni, & Mani, 2016; Mascarelli, McQuillan, Harms, Harms, & Breitschwerdt, 2014), and it is conceivable that the bacteria originate from the birds, rather than from the mites. That would suggest that the host range for *Bartonella* spp. is broader than previously reported and here we expand the list of potential sources for this zoonotic infection. However, Bartonellaceae can be symbiotic in other hosts, such as honey bees and ants (Segers, Kešnerová, Kosoy, & Engel, 2017, Bisch et al. 2018). Further, *Bartonella‐like* symbionts were recently found in a number astigmatid mites (Kopecký, Nesvorná, & Hubert, 2014), indicating that the *Bartonella* detected here might be quill mite symbionts, rather than pathogens. With our data, it is not possible to rule out either possibility.

Finally, we found the symbionts *Spiroplasma* and *Wolbachia* in quill mites. Both of these are common across a range of arthropod species (Anbutsu & Fukatsu, 2011; Zug & Hammerstein, 2012), are typically transmitted intraovarially, and may cause sex ratio distorting phenotypes (Haselkorn, 2010; Werren, 1997). Whereas *Spiroplasma* was so far not reported from quill mites, *Wolbachia* was previously detected and our findings confirm that this is a common symbiont of quill mites (Glowska et al., 2015). The observed presence and abundance of both taxa are not uniform across the sampled taxa (Figures 1, 4a). For example, *Wolbachia* is most abundant in mites parasitizing birds of the genera *Turdus, Erithacus,* and *Fringilla*, whereas *Spiroplasma* is most strongly associated with mites parasitizing *Carduelis*. One reason for this may be that some taxa are more susceptible than others for endosymbiosis with certain bacteria, and this phylogenetic effect has been reported for other host taxa as well (Gerth, Saeed, White, & Bleidorn, 2015; Russell, 2012). Strikingly, very high *Spiroplasma* abundances were only found in two investigated mite species that are specialized parasites of two bird species of the genus *Carduelis* (Figure 4a., Table 1). A number of samples showed very low *Spiroplasma* titers, which may be a result of genuine low titer infections or stem from contamination via simultaneously processed libraries (e.g., through index hopping, Kircher, Sawyer, & Meyer, 2012). For the samples with unambiguously high *Spiroplasma* titers, the bird host phylogeny seems to be the best predictor for a *Spiroplasma* infection. One interpretation of this pattern is a history of horizontal transmission of the symbiont via the bird hosts. Horizontal transfers have been inferred from phylogenetic data for *Wolbachia* and *Spiroplasma* previously (Gerth, Röthe, & Bleidorn, 2013; Haselkorn, Markow, & Moran, 2009), and for both symbionts, horizontal transmissions were also demonstrated experimentally (Huigens et al., 2000; Jaenike, Polak, Fiskin, Helou, & Minhas, 2007). Although the potential mechanism of horizontal symbiont transmission via feather quills is unclear, our data suggest that the bird–parasite interactions may be important for endosymbiont transmission dynamics in quill mites.

Interestingly, we found that *Spiroplasma* presence leads to reduced *Wolbachia* titers, although this is based on a small sample size for samples that are both *Wolbachia* and *Spiroplasma* positive (*N* = 11, Figure 4b). Furthermore, in these eleven samples, *Spiroplasma* and *Wolbachia* titers seem to be positively correlated (Figure 4c). It is conceivable that sharing of hosts leads to competition for finite resources the host can provide (Vautrin & Vavre, 2009), and thus the growth of one symbiont might limit that of another. In *Drosophila,* for example, *Spiroplasma* seem to limit the proliferation of *Wolbachia* (Goto, Anbutsu, & Fukatsu, 2006) and in aphids, competition between co‐occurring secondary symbionts appears to be harmful to the host (Oliver et al. 2006). Such negative fitness impacts can also be expected when both symbiont titers are very high, as found here in quill mites. Although purely speculative, this may be the reason why we only observed simultaneously high *Spiroplasma* and *Wolbachia* titers in very few of the 126 investigated quill mites (Figure 4c).

## 5 SUMMARY

We found a diverse, but relatively uniform set of bacterial taxa within quill mites that includes arthropod endosymbionts, pathogens, and bird‐associated bacteria. The importance of most of these microbes for quill mite biology is unclear, but abundances and distribution patterns suggest that *Spiroplasma* and *Wolbachia* are the most important quill mite associates.

## CONFLICT OF INTEREST

None declared.

## AUTHOR CONTRIBUTIONS

EG, ZF, and MG involved in study conception and design. EG involved in collecting and identification of the mite material, funding acquisition, project administration, supervision, writing review and editing and contributed resources. ZF and MD involved in acquisition of data. MG analyzed the data. EG and MG interpreted the data and wrote the paper.

## ETHICAL STATEMENT

None required.

## Data Availability

The data that support the findings of this study are openly available under NCBI BioProject accession number PRJNA482380 and in Zenodo repository at ://doi.org/10.5281/zenodo.3475151.

## APPENDIX 1

**Table A1** List of all ASVs detected in this study, ordered by abundance (relative abundances summed over all samples) available at http://doi.org/10.5281/zenodo.3475151

## References

[mbo3964-bib-0001] Anbutsu, H. , & Fukatsu, T. (2011). *Spiroplasma* as a model insect endosymbiont. Environmental Microbiology Reports, 3, 144–153. 10.1111/j.1758-2229.2010.00240.x 23761245

[mbo3964-bib-0002] Azad, A. F. , & Beard, C. B. (1998). Rickettsial pathogens and their arthropod vectors. Emerging Infectious Diseases, 4, 179 10.3201/eid0402.980205 9621188 PMC2640117

[mbo3964-bib-0003] Ballinger, M. , & Perlman, S. (2017). Generality of toxins in defensive symbiosis: Ribosome‐inactivating proteins and defense against parasitic wasps in *Drosophila* . PLoS Path, 13, e1006431 10.1371/journal.ppat.1006431 PMC5500355 28683136

[mbo3964-bib-0004] Benjamini, Y. , & Hochberg, Y. (1995). Controlling the false discovery rate: A practical and powerful approach to multiple testing. Journal of the Royal Statistical Society Series B, 57, 289–300.

[mbo3964-bib-0005] Billeter, S. , Levy, M. , Chomel, B. , & Breitschwerdt, E. (2008). Vector transmission of *Bartonella* species with emphasis on the potential for tick transmission. Medical and Veterinary Entomology, 22, 1–15. 10.1111/j.1365-2915.2008.00713.x 18380649

[mbo3964-bib-0006] Bisch, G. , Neuvonen, M. M. , Pierce, N. E. , Russell, J. A. , Koga, R. , … Andersson, S. G. E. (2018). Genome evolution of bartonellaceae symbionts of ants at the opposite ends of the trophic scale. Genome Biology and Evolution, 10(7), 1687–1704. 10.1093/gbe/evy126 29982531 PMC6044324

[mbo3964-bib-0007] Callahan, B. J. , McMurdie, P. J. , Rosen, M. J. , Han, A. W. , Johnson, A. J. A. , & Holmes, S. P. (2016). DADA2: High‐resolution sample inference from Illumina amplicon data. Nature Methods, 13, 581–583. 10.1038/nmeth.3869 27214047 PMC4927377

[mbo3964-bib-0008] Casto, S. D. (1974a). A nocturnal dispersal rhythm in the quill mite, *Syringophiloidus minor* (Berlese) (Prostigmata: Syringophilidae). Journal of Medical Entomology, 11, 113–114. 10.1093/jmedent/11.1.113 4857200

[mbo3964-bib-0009] Casto, S. D. (1974b). Entry and exit of syringophilid mites (Acarina: Syringophilidae) from the lumen of the quill. The Wilson Bulletin, 86, 272–278.

[mbo3964-bib-0010] Chan, T. F. , Ji, K. M. , Yim, A. K. , Liu, X. Y. , Zhou, J. W. ,… Tsui, S. K. W. (2015). The draft genome, transcriptome, and microbiome of *Dermatophagoides farinae* reveal a broad spectrum of dust mite allergens. Journal of Allergy and Clinical Immunology, 135, 539–548. 10.1016/j.jaci.2014.09.031 25445830

[mbo3964-bib-0011] Colston, T. J. , & Jackson, C. R. (2016). Microbiome evolution along divergent branches of the vertebrate tree of life: What is known and unknown. Molecular Ecology, 25, 3776–3800. 10.1111/mec.13730 27297628

[mbo3964-bib-0012] R Core Team (2015). A language and environment for statistical computing. Vienna, Austria: (R Foundation for Statistical Computing.

[mbo3964-bib-0013] Dabert, J. , Ehrnsberger, R. , & Dabert, M. (2008). *Glaucalges tytonis* sp. n. (Analgoidea, Xolalgidae) from the barn owl *Tyto alba *(Strigiformes, Tytonidae): Compiling morphology with DNA barcode data for taxon descriptions in mites (Acari). Zootaxa, 1719, 41–52.

[mbo3964-bib-0014] Davis, N. M. , Proctor, D. M. , Holmes, S. P. , Relman, D. A. & Callahan B. J. (2018). Simple statistical identification and removal of contaminant sequences in marker-gene and metagenomics data. Microbiome, 6, 226 10.1186/s40168-018-0605-2 30558668 PMC6298009

[mbo3964-bib-0015] De Goffau, M. C. , Lager, S. , Salter, S. J. , Wagner, J. , Kronbichler, A. , Charnock‐Jones, D. S. , … Parkhill, J. (2018). Recognizing the reagent microbiome. Nature Microbiology, 3, 851–853. 10.1038/s41564-018-0202-y 30046175

[mbo3964-bib-0016] Dille, J. W. , Rogers, C. M. , & Schneegurt, M. A. (2016). Isolation and characterization of bacteria from the feathers of wild Dark‐eyed Juncos (*Junco hyemalis*). The Auk, 133, 155–167.

[mbo3964-bib-0017] Dixon, P. (2003). VEGAN, a package of R functions for community ecology. Journal of Vegetation Science, 14, 927–930. 10.1111/j.1654-1103.2003.tb02228.x

[mbo3964-bib-0018] Duron, O. , Morel, O. , Noël, V. , Buysse, M. , Binetruy, F. , Lancelot, R. , … Vial, L. (2018). Tick‐bacteria mutualism depends on B vitamin synthesis pathways. Current Biology, 28, 1896–1902.e5. 10.1016/j.cub.2018.04.038 29861133

[mbo3964-bib-0019] Ebani, V. V. , Bertelloni, F. , & Mani, P. (2016). Molecular survey on zoonotic tick‐borne bacteria and chlamydiae in feral pigeons (*Columba livia domestica*). Asian Pacific Journal of Tropical Medicine, 9, 324–327. 10.1016/j.apjtm.2016.03.005 27086148

[mbo3964-bib-0020] Engel, K. , Sauer, J. , Jünemann, S. , Winkler, A. , Wibberg, D. , Kalinowski, J. , … Caspers, B. A. (2018). Individual‐ and species‐specific skin microbiomes in three different estrildid finch species revealed by 16S amplicon sequencing. Microbial Ecology, 76, 518–529. 10.1007/s00248-017-1130-8 29282519

[mbo3964-bib-0021] Erban, T. , Klimov, P. B. , Smrz, J. , Phillips, T. W. , Nesvorna, M. , Kopecky, J. , & Hubert, J. (2016). Populations of stored product mite tyrophagus putrescentiae differ in their bacterial communities. Frontiers in Microbiology, 7, 1046 10.3389/fmicb.2016.01046 27462300 PMC4940368

[mbo3964-bib-0022] Gerth, M. , Röthe, J. , & Bleidorn, C. (2013). Tracing horizontal *Wolbachia* movements among bees (Anthophila): A combined approach using MLST data and host phylogeny. Molecular Ecology, 22, 6149–6162. 24118435 10.1111/mec.12549

[mbo3964-bib-0023] Gerth, M. , Saeed, A. , White, J. A. , & Bleidorn, C. (2015). Extensive screen for bacterial endosymbionts reveals taxon‐specific distribution patterns among bees (Hymenoptera, Anthophila). FEMS Microbiology Ecology, 91, fiv047 10.1093/femsec/fiv047 25914139

[mbo3964-bib-0024] Glowska, E. , Dragun‐Damian, A. , Dabert, M. , & Gerth, M. (2015). New *Wolbachia* supergroups detected in quill mites (Acari: Syringophilidae). Infection, Genetics and Evolution, 30, 140–146. 10.1016/j.meegid.2014.12.019 25541519

[mbo3964-bib-0025] Goto, S. , Anbutsu, H. , & Fukatsu, T. (2006). Asymmetrical interactions between *Wolbachia* and *Spiroplasma* endosymbionts coexisting in the same insect host. Applied and Environmental Microbiology, 72, 4805–4810. 10.1128/AEM.00416-06 16820474 PMC1489378

[mbo3964-bib-0026] Hammer, T. J. , Janzen, D. H. , Hallwachs, W. , Jaffe, S. P. , & Fierer, N. (2017). Caterpillars lack a resident gut microbiome. Proceedings of the National Academy of Sciences of the United States of America, 114, 9641–9646. 10.1073/pnas.1707186114 28830993 PMC5594680

[mbo3964-bib-0027] Haselkorn, T. S. (2010). The *Spiroplasma* heritable bacterial endosymbiont of *Drosophila* . Fly, 4, 80–87. 10.4161/fly.4.1.10883 20081357

[mbo3964-bib-0028] Haselkorn, T. S. , Markow, T. A. , & Moran, N. A. (2009). Multiple introductions of the *Spiroplasma* bacterial endosymbiont into *Drosophila* . Molecular Ecology, 18, 1294–1305. 19226322 10.1111/j.1365-294X.2009.04085.x

[mbo3964-bib-0029] Hird, S. M. (2017). Evolutionary biology needs wild microbiomes. Frontiers in Microbiology, 8, 725 10.3389/fmicb.2017.00725 28487687 PMC5404107

[mbo3964-bib-0030] Hogg, J. , & Lehane, M. (1999). Identification of bacterial species associated with the sheep scab mite (*Psoroptes ovis*) by using amplified genes coding for 16S rRNA. Applied and Environmental Microbiology, 65, 4227–4229. 10473440 10.1128/aem.65.9.4227-4229.1999 PMC99765

[mbo3964-bib-0031] Hosokawa, T. , Koga, R. , Kikuchi, Y. , Meng, X.‐Y. , & Fukatsu, T. (2010). *Wolbachia* as a bacteriocyte‐associated nutritional mutualist. Proceedings of the National Academy of Sciences of the United States of America, 107, 769–774. 10.1073/pnas.0911476107 20080750 PMC2818902

[mbo3964-bib-0032] Hubert, J. , Erban, T. , Kopecky, J. , Sopko, B. , Nesvorna, M. , Lichovnikova, M. , … Sparagano, O. (2017). Comparison of microbiomes between red poultry mite populations (*Dermanyssus gallinae*): Predominance of *Bartonella*‐like bacteria. Microbial Ecology, 74, 947–960. 10.1007/s00248-017-0993-z 28534089

[mbo3964-bib-0033] Hubert, J. , Kamler, M. , Nesvorna, M. , Ledvinka, O. , Kopecky, J. , & Erban, T. (2016). Comparison of *Varroa destructor* and worker honeybee microbiota within hives indicates shared bacteria. Microbial Ecology, 72, 448–459. 10.1007/s00248-016-0776-y 27129319

[mbo3964-bib-0034] Hubert, J. , Kopecky, J. , Nesvorna, M. , Perotti, M. A. , & Erban, T. (2016). Detection and localization of *Solitalea*‐like and *Cardinium* bacteria in three *Acarus siro* populations (Astigmata: Acaridae). Experimental and Applied Acarology, 70, 309–327. 10.1007/s10493-016-0080-z 27502113

[mbo3964-bib-0035] Hubert, J. , Nesvorna, M. , Klimov, P. , Dowd, S. E. , Sopko, B. , & Erban, T. (2019). Differential allergen expression in three *Tyrophagus putrescentiae* strains inhabited by distinct microbiome. Allergy, 10.1111/all.13921. Epub ahead of print. 31121066

[mbo3964-bib-0036] Huigens, M. E. , Luck, R. F. , Klaassen, R. H. G. , Maas, M. , Timmermans, M. , & Stouthamer, R. (2000). Infectious parthenogenesis. Nature, 405, 178–179. 10.1038/35012066 10821272

[mbo3964-bib-0037] Jaenike, J. , Polak, M. , Fiskin, A. , Helou, M. , & Minhas, M. (2007). Interspecific transmission of endosymbiotic *Spiroplasma* by mites. Biology Letters, 3, 23–25. 17443956 10.1098/rsbl.2006.0577 PMC2373825

[mbo3964-bib-0038] Jetz, W. , Thomas, G. H. , Joy, J. B. , Hartmann, K. , & Mooers, A. O. (2012). The global diversity of birds in space and time. Nature, 491, 444–448. 10.1038/nature11631 23123857

[mbo3964-bib-0039] Ji, B. , & Nielsen, J. (2015). From next‐generation sequencing to systematic modeling of the gut microbiome. Frontiers in Genetics, 6, 219 10.3389/fgene.2015.00219 26157455 PMC4477173

[mbo3964-bib-0040] Kethley, J. (1970). A revision of the family Syringophilidae (Prostigmata: Acarina). Contributions of the American Entomological Institute, 5, 1–76.

[mbo3964-bib-0041] Kethley, J. (1971). Population regulation in quill mites (Acarina: Syringophilidae). Ecology, 52, 1113–1118. 10.2307/1933821

[mbo3964-bib-0042] King, K. C. , Brockhurst, M. A. , Vasieva, O. , Paterson, S. , Betts, A. , Ford, S. A. , … Hurst, D. D. G. (2016). Rapid evolution of microbe‐mediated protection against pathogens in a worm host. The ISME Journal, 10, 1915–1924. 10.1038/ismej.2015.259 26978164 PMC5029159

[mbo3964-bib-0043] Kircher, M. , Sawyer, S. , & Meyer, M. (2012). Double indexing overcomes inaccuracies in multiplex sequencing on the Illumina platform. Nucleic Acids Research, 40, e3 10.1093/nar/gkr771 22021376 PMC3245947

[mbo3964-bib-0044] Klangthong, K. , Promsthaporn, S. , Leepitakrat, S. , Schuster, A. L. , McCardle, P. W. , Kosoy, M. , & Takhampunya, R. (2015). The distribution and diversity of *Bartonella* species in rodents and their ectoparasites across Thailand. PLoS One, 10, e0140856 10.1371/journal.pone.0140856 26484537 PMC4617648

[mbo3964-bib-0045] Kopecký, J. , Nesvorná, M. , & Hubert, J. (2014). *Bartonella*‐like bacteria carried by domestic mite species. Experimental and Applied Acarology, 64, 21–32. 10.1007/s10493-014-9811-1 24711066

[mbo3964-bib-0046] Kwong, W. K. , Medina, L. A. , Koch, H. et al. (2017). Dynamic microbiome evolution in social bees. Science Advances, 3, e1600513 10.1126/sciadv.1600513 28435856 PMC5371421

[mbo3964-bib-0047] Laughton, A. M. , Fan, M. H. , & Gerardo, N. M. (2013). The combined effects of bacterial symbionts and aging on life history traits in the pea aphid, *Acyrthosiphon pisum* . Applied and Environmental Microbiology, 80, 470–477. 10.1128/AEM.02657-13 24185857 PMC3911086

[mbo3964-bib-0048] Łukasik, P. , Newton, J. A. , Sanders, J. G. , Hu, Y. , Moreau, C. S. , Kronauer, D. J. C. , … Russell, J. A. (2017). The structured diversity of specialized gut symbionts of the New World army ants. Molecular Ecology, 26, 3808–3825. 10.1111/mec.14140 28393425

[mbo3964-bib-0049] Makepeace, B.L. , Gill, A.C. (2016) Wolbachia In: Thomas S., (Eds). Rickettsiales. Cham, Switzerland: Springer.

[mbo3964-bib-0050] Martin, M. (2011). Cutadapt removes adapter sequences from high‐throughput sequencing reads. EMBnet.journal, 17, 10–12.

[mbo3964-bib-0051] Mascarelli, P. E. , McQuillan, M. , Harms, C. A. , Harms, R. V. , & Breitschwerdt, E. B. (2014). *Bartonella henselae* and *B. koehlerae* DNA in birds. Emerging Infectious Diseases, 20, 490–492. 24589235 10.3201/eid2003.130563 PMC3944876

[mbo3964-bib-0052] McFall‐Ngai, M. , Hadfield, M. G. , Bosch, T. C. , Carey, H. V. , Domazet‐Lošo, T. , Douglas, A. E. et al. (2013). Animals in a bacterial world, a new imperative for the life sciences. Proceedings of the National Academy of Sciences of the United States of America, 110, 3229–3236. 10.1073/pnas.1218525110 23391737 PMC3587249

[mbo3964-bib-0053] McMurdie, P. J. , & Holmes, S. (2013). Phyloseq: An R package for reproducible interactive analysis and graphics of microbiome census data. PLoS ONE, 8, e61217 10.1371/journal.pone.0061217 23630581 PMC3632530

[mbo3964-bib-0054] Moro, C. V. , Thioulouse, J. , Chauve, C. , & Zenner, L. (2011). Diversity, geographic distribution, and habitat‐specific variations of microbiota in natural populations of the chicken mite, *Dermanyssus gallinae* . Journal of Medical Entomology, 48, 788–796. 10.1603/ME10113 21845937

[mbo3964-bib-0055] Neglia, G. , Veneziano, V. , De Carlo, E. , Galiero, G. , Borriello, G. , Francillo, M. , … Manna, L. (2013). Detection of *Brucella abortus* DNA and RNA in different stages of development of the sucking louse *Haematopinus tuberculatus* . BMC Veterinary Research, 9, 236 10.1186/1746-6148-9-236 24289112 PMC4220825

[mbo3964-bib-0056] Oh, H. , Ishii, A. , Tongu, Y. , & Itano, K. (1986). Microorganisms associated with the house‐dust mite Dermatophagoides. Medical Entomology and Zoology, 37, 229–235. 10.7601/mez.37.229

[mbo3964-bib-0057] Oliver, K. M. , Moran, N. A. , & Hunter, M. S. (2006). Costs and benefits of a superinfection of facultative symbionts in aphids. Proceedings of the Royal Society B: Biological Sciences, 273(1591), 1273–1280. 10.1098/rspb.2005.3436 PMC1560284 16720402

[mbo3964-bib-0058] Pappas, G. , Papadimitriou, P. , Akritidis, N. , Christou, L. , & Tsianos, E. V. (2006). The new global map of human brucellosis. The Lancet Infectious Diseases, 6, 91–99. 10.1016/S1473-3099(06)70382-6 16439329

[mbo3964-bib-0059] Pearce, D. S. , Hoover, B. A. , Jennings, S. , Nevitt, G. A. , & Docherty, K. M. (2017). Morphological and genetic factors shape the microbiome of a seabird species (*Oceanodroma leucorhoa*) more than environmental and social factors. Microbiome, 5, 146 10.1186/s40168-017-0365-4 29084611 PMC5663041

[mbo3964-bib-0060] Pritulin, P. (1954). On the transmission of brucellosis by the pasture ticks *Dermacentor nuttallia* and *Hyalomma marginatum* . Veterinariya, 31, 31–33.

[mbo3964-bib-0061] Quast, C. , Pruesse, E. , Yilmaz, P. , Gerken, J. , Schweer, T. , Yarza, P. , … Glöckner, F. O. (2013). The SILVA ribosomal RNA gene database project: Improved data processing and web‐based tools. Nucleic Acids Research, 41, D590–D596. 10.1093/nar/gks1219 23193283 PMC3531112

[mbo3964-bib-0062] Reeves, W. K. , Nelder, M. P. , Cobb, K. D. , & Dasch, G. A. (2006). *Bartonella* spp. in deer keds, *Lipoptena mazamae* (Diptera: Hippoboscidae), from Georgia and South Carolina, USA. Journal of Wildlife Diseases, 42, 391–396. 10.7589/0090-3558-42.2.391 16870863

[mbo3964-bib-0063] Rementzova, M. (1966).Parasitical arthropods vectors of the brucellic infectionIn Corradetti A. (Eds.), Proceedings of the first international congress of parasitology (pp. 147–150). Oxford, UK: Pergamon.

[mbo3964-bib-0064] Russell, J. A. (2012). The ants (*Hymenoptera*: Formicidae) are unique and enigmatic hosts of prevalent *Wolbachia* (Alphaproteobacteria) symbionts. Myrmecological News, 16, 7–23.

[mbo3964-bib-0065] Salter, S. J. , Cox, M. J. , Turek, E. M. , Calus, S. T. , Cookson, W. O. , Moffatt, M. F. , … Walker, A. W. (2014). Reagent and laboratory contamination can critically impact sequence‐based microbiome analyses. BMC Biology, 12, 87 10.1186/s12915-014-0087-z 25387460 PMC4228153

[mbo3964-bib-0066] Sboner, A. , Mu, X. , Greenbaum, D. , Auerbach, R. K. , & Gerstein, M. B. (2011). The real cost of sequencing: Higher than you think!. Genome Biology, 12, 125 10.1186/gb-2011-12-8-125 21867570 PMC3245608

[mbo3964-bib-0067] Segers, F. H. I. D. , Kešnerová, L. , Kosoy, M. , & Engel, P. (2017). Genomic changes associated with the evolutionary transition of an insect gut symbiont into a blood‐borne pathogen. The ISME Journal, 11, 1232–1244. 10.1038/ismej.2016.201 28234349 PMC5437933

[mbo3964-bib-0068] Sharon, G. , Segal, D. , Ringo, J. , Hefetz, A. , Zilber‐Rosenberg, I. , & Rosenberg, E. (2010). Commensal bacteria play a role in mating preference of *Drosophila* *melanogaster* . Proceedings of the National Academy of Sciences of the United States of America, 107, 20051–20056. 10.1073/pnas.1009906107 21041648 PMC2993361

[mbo3964-bib-0069] Skoracki, M. , Michalik, J. , Skotarczak, B. , Rymaszewska, A. , Sikora, B. , Hofman, T. , … Sawczuk, M. (2006). First detection of *Anaplasma phagocytophilum* in quill mites (Acari: Syringophilidae) parasitizing passerine birds. Microbes and Infection, 8, 303–307. 10.1016/j.micinf.2005.06.029 16293433

[mbo3964-bib-0070] Skoracki, M. , Spicer, G. S. , & Oconnor, B. M. (2016). A systematic review of the subfamily Syringophilinae (Acari: Syringophilidae) of the Nearctic region. Part 1: Quill mites associated with passerines (Aves: Passeriformes). Zootaxa, 4084, 451. 27394276 10.11646/zootaxa.4084.4.1

[mbo3964-bib-0071] Therese, K. L. , Anand, A. R. , & Madhavan, H. N. (1998). Polymerase chain reaction in the diagnosis of uveitis. British Journal of Ophthalmology, 82, 1078–1082. 9893601 10.1136/bjo.82.9.1078 PMC1722759

[mbo3964-bib-0072] Valerio, C. , Murray, P. , Arlian, L. , & Slater, J. (2005). Bacterial 16S ribosomal DNA in house dust mite cultures. Journal of Allergy and Clinical Immunology, 116, 1296–1300. 10.1016/j.jaci.2005.09.046 16337462

[mbo3964-bib-0073] Vautrin, E. , & Vavre, F. (2009). Interactions between vertically transmitted symbionts: Cooperation or conflict? Trends in Microbiology, 17, 95–99. 19230673 10.1016/j.tim.2008.12.002

[mbo3964-bib-0074] Werren, J. H. (1997). Biology of *Wolbachia* . Annual Review of Entomology, 42, 587–609. 10.1146/annurev.ento.42.1.587 15012323

[mbo3964-bib-0075] Wickham, H. (2009). ggplot2: Elegant graphics for data analysis. New York, NY: Springer‐Verlag.

[mbo3964-bib-0076] Young, E. J. (2006). Brucella sppIn Gillespie S. H., & Hawkey P. M. (Eds.), Principles and practice of clinical bacteriology (pp. 265–271). Chichester, UK: John Wiley and Sons Ltd.

[mbo3964-bib-0077] Zheludkov, M. , & Tsirelson, L. (2010). Reservoirs of *Brucella* infection in nature. Biology Bulletin, 37, 709–715. 10.1134/S106235901007006X

[mbo3964-bib-0078] Zug, R. , & Hammerstein, P. (2012). Still a host of hosts for *Wolbachia*: Analysis of recent data suggests that 40% of terrestrial arthropod species are infected. PLoS One, 7, e38544 10.1371/journal.pone.0038544 22685581 PMC3369835

